# Cross-Talking Pathways of Forkhead Box O1 (FOXO1) Are Involved in the Pathogenesis of Alzheimer's Disease and Huntington's Disease

**DOI:** 10.1155/2022/7619255

**Published:** 2022-02-03

**Authors:** Liyang Liu, Jun Bai, Fangxi Liu, Ying Xu, Mei Zhao, Chuansheng Zhao, Zhike Zhou

**Affiliations:** ^1^Department of Neurology, The First Affiliated Hospital, China Medical University, Shenyang, 110001 Liaoning, China; ^2^Cancer Systems Biology Center, The China-Japan Union Hospital, Jilin University, Changchun, 130033 Jilin, China; ^3^Computational Systems Biology Lab, Department of Biochemistry and Molecular Biology and Institute of Bioinformatics, The University of Georgia, USA; ^4^Department of Cardiology, The Shengjing Affiliated Hospital, China Medical University, Shenyang, 110004 Liaoning, China; ^5^Department of Geriatrics, The First Affiliated Hospital, China Medical University, Shenyang, 110001 Liaoning, China

## Abstract

Alzheimer's disease (AD) and Huntington's disease (HD) are destructive worldwide diseases. Efforts have been made to elucidate the process of these two diseases, yet the pathogenesis remains elusive as it involves a combination of multiple factors, including genetic and environmental ones. To explore the potential role of forkhead box O1 (FOXO1) in the development of AD and HD, we identified 1,853 differentially expressed genes (DEGs) from 19,414 background genes in both the AD&HD/control and FOXO1-low/high groups. Four coexpression modules were predicted by the weighted gene coexpression network analysis (WGCNA), among which blue and turquoise modules had the strongest correlation with AD&HD and high expression of FOXO1. Functional enrichment analysis showed that DEGs in these modules were enriched in phagosome, cytokine-cytokine receptor interaction, cellular senescence, FOXO signaling pathway, pathways of neurodegeneration, GABAergic synapse, and AGE-RAGE signaling pathway in diabetic complications. Furthermore, the cross-talking pathways of FOXO1 in AD and HD were jointly determined in a global regulatory network, such as the FOXO signaling pathway, cellular senescence, and AGE-RAGE signaling pathway in diabetic complications. Based on the performance evaluation of the area under the curve of 85.6%, FOXO1 could accurately predict the onset of AD and HD. We then identified the cross-talking pathways of FOXO1 in AD and HD, respectively. More specifically, FOXO1 was involved in the FOXO signaling pathway and cellular senescence in AD; correspondingly, FOXO1 participated in insulin resistance, insulin, and the FOXO signaling pathways in HD. Next, we use GSEA to validate the biological processes in AD&HD and FOXO1 expression. In GSEA analysis, regulation of protein maturation and regulation of protein processing were both enriched in the AD&HD and FOXO1-high groups, suggesting that FOXO1 may have implications in onset and progression of these two diseases through protein synthesis. Consequently, a high expression of FOXO1 is a potential pathogenic factor in both AD and HD involving mechanisms of the FOXO signaling pathway, AGE-RAGE signaling pathway in diabetic complications, and cellular senescence. Our findings provide a comprehensive perspective on the molecular function of FOXO1 in the pathogenesis of AD and HD.

## 1. Introduction

Neurodegenerative disease (ND), a heterogeneous group of devastating and irreversible disorders, is characterized by a progressive loss of neurons due to the deposition of isomerism proteins, such as amyloid-beta (A*β*), prion, huntingtin protein (HTT), tau, and alpha-synuclein [1, 2]. With the extension of life expectancy in general population, the incidence of ND is on the rise. Herein, our study focused on two types of NDs, namely, Alzheimer's disease (AD) and Huntington's disease (HD). As the most common cause of dementia worldwide, AD is pathologically characterized by deposition of A*β* plaques composed of amyloid-beta protein and neurofibrillary tangles (NFTs) consisting of hyperphosphorylated tau protein, which are attributed to the overproduction and impaired clearance [3, 4]. The main manifestation of HD is motor impairment and cognitive deficit derived from neuronal dysfunction and death, which is due to the toxicity of the expansion of the polyglutamine region in the HTT protein as a consequence of the mutated gene [5, 6]. Of note, these two diseases are associated with aberrant proteins. Since there is no cure for either condition, efforts are under way to halt or even prevent them by studying genetic factors along with their underlying mechanisms in aberrant protein metabolism. So far, multiple mechanisms have been found to be involved in the occurrence and development of AD and HD [7].

FOXO belongs to the family of transcription factor forkhead box O genes with four isoforms (i.e., FOXO1, FOXO3a, FOXO4, and FOXO6), all of which share highly conserved domains [8]. The functions of FOXO proteins are thought to regulate the expression of genes during biological events including apoptosis, cell cycle control, glucose metabolism, antioxidative stress, and life longevity. Hence, dysfunction of FOXOs leads to diseases and conditions involving shorten life span, cancer, metabolic diseases, immune system disorders, and ND [9–11]. The processes of phosphorylation through the PI3K/Akt, JNK/c-Jun, or AMPK pathway in response to growth factors, insulin/IGF-1, oxidative, and nutrient stress are the main regulatory mechanisms of FOXO proteins. Although all the four isoforms have highly conserved domains and overlapping functions to certain extent [12], additional evidence shows that different isoforms of FOXO regulate a nonredundant set of genes [13]. For instance, FOXO1 plays critical roles in the processes of energy metabolism, longevity, cell cycle arrest, and cellular senescence. Suppression of FOXO1 protein by insulin/IGF-1 or growth factors through the PI3K/Akt pathway decreases its transcriptional function to mediate nutrient metabolism against food deprivation and energy deficiency. Dysfunction of such pathway could lead to metabolic diseases including diabetes, insulin resistance, and increase of food intake [14]. Moreover, FOXO1 mediates cell cycle arrest and apoptosis via the JNK/c-Jun pathway, which has been observed in cells under the condition of growth factor deprivation or oxidative stress [15–17]. With the presence of reactive oxygen species (ROS) or energy deficiency, FOXO1 is activated via the AMPK pathway to induce autophagy, an important mechanism for the clearance of abnormal proteins and organelles; conversely, this process is inhibited by activation of the PI3K/Akt pathway in nonneuronal systems [18, 19]. One plausible interpretation is that activation of FOXO1 protein induces neuronal loss, resulting in persistent neurodegeneration [20]. Although the regulatory role of FOXO1 proteins has been investigated, few studies have focused on the coordinated mechanism of FOXO1-related pathways in the development of AD and HD. Accordingly, we performed a comprehensive genomic analysis of FOXO1 based on gene expression data and functional annotations with the aim of illuminating the common underlying role of FOXO1 in the pathogenesis of AD and HD.

## 2. Materials and Methods

### 2.1. Data Processing

We downloaded the RNA gene expression profiles of GSE33000 from the Gene Expression Omnibus (GEO) database, which included 467 patients with neurodegenerative diseases (310 AD and 157 HD) and 157 nondementia controls [21]. We then selected a total of 465 patients and controls over 60 years of age, including 367 patients (305 AD and 62 HD) and 98 controls for analysis. The *normalizeBetweenArrays* function in the *limma* package of R software (version 3.6.2) was used to normalize the gene expression profile [22]. Based on clinical data recorded in previous studies, the age and gender between cases and controls were matched [23, 24]. The mean age of AD and HD was 79.24 ± 9.11 years (range: 60-100 years), and the mean age of nondementia was 69.06 ± 7.70 years (range: 60-106 years). The enrolled samples were divided into the FOXO1-high and low groups by defining the mean expression value of FOXO1 as the cut-off point.

### 2.2. Identification of Differentially Expressed Genes (DEGs)

DEGs were generated in the AD&HD/control and FOXO1-high/low groups using the *lmFit* and *eBayes* functions of *limma* package in R software. The fold changes (FC) in the expression of individual genes were calculated, with ∣logFC | >0.15 and false discovery rate- (FDR-) adjusted *P* < 0.05 considered thresholds [25].

### 2.3. Coexpression Network Construction and Functional Enrichment Analysis of Coexpressed Modules

Using the *WGCNA* package in R software, the gene coexpression network analysis was constructed by clustering overlapping DEGs between the AD&HD/control and FOXO1-high/low groups into multiple functional modules. Weight gene correlation network analysis (WGCNA), an advanced data exploration technique, not only allows the construction of interconnected node modules that represent network-based data volumes and alleviate the problem of multiple testing but also screens for modules that are relevant to clinical traits [26]. The *hclust* function was used to eliminate outliers from the samples. A *pickSoftThreshold* function was used to determine the appropriate power value of 16 when the degree of independence was set to 0.8. To ensure high reliability of the results, each module contained at least 30 genes. From thousands of genes, interesting modules of DEGs were identified by WGCNA, and then, the Kyoto Encyclopedia of Genes Genomes (KEGG) pathway analysis was performed on the genes in each module using the *ClusterProfiler* package in R software. FDR adjusted *P* < 0.05 was used as the threshold to define the significant differences in biological functions and enrichment pathways enriched in each module.

### 2.4. Construction of Module-Pathway Network of FOXO1

Correlations between intramodular connectivity and clinical phenotype were used to estimate module-pathway associations, so that highly phenotypically related expression modules could be readily identified. Gene significance (GS) was calculated as the absolute value of the correlation between the gene expression profile and each trait; module membership (MM) was defined as the correlation between the gene expression profiles in each module. Scatterplot of GS vs. MM in each module was plotted using the *verboseScatterplot* function to represent the correlation between intramodular connectivity and clinical trait. In modules related to the trait of interest, genes with higher module membership tend to have higher genetic significance and biological importance [27]. The global regulatory network of module genes with the highest interest was constructed using the Search Tool for the Retrieval of Interacting Genes (STRING; http://STRING-db.org) online database [28], in which cross-talking pathways of FOXO1 were annotated by the functional enrichment analysis of the KEGG pathway. The visualization of global regulatory network and cross-talking pathways of FOXO1 were accomplished by employing Cytoscape software [29].

### 2.5. Analysis of the Area under the Curve (AUC)

Adopting the *pROC* package, AUC analysis was performed to predict the diagnostic performance of FOXO1 in differentiation between AD&HD and controls. Bilateral *P* value of less than 0.05 was considered statistically significant.

### 2.6. Gene Set Enrichment Analysis (GSEA)

According to the median expression of FOXO1, samples were divided into the FOXO1-high and low expression groups. After normalization of the gene expression profile, GSEA was performed to screen for the biological process of Gene Ontology (GO) terms in the AD&HD and FOXO1-high groups [30]. The threshold of significant enrichment was obtained based on the permutation test (the number of permutations was set to 1000, with a *P* value less than 0.05) applying default weighting statistic for each parameter. Enriched data in GSEA analysis was visualized using packages of *ClusterProfler*, *ggplot2*, *enrichplot*, and *GSEABase*.

### 2.7. Workflow

**Figure 1:**
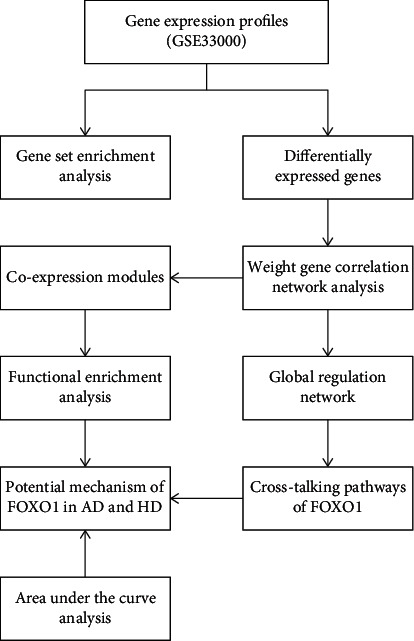
The workflow diagram of the present study. AD: Alzheimer's disease; HD: Huntington's disease.

To investigate the functions of FOXO1 in the pathogenesis of AD and HD, we conducted systematic analysis following the steps in Figure 1. Differential expression analysis of genes was conducted on basis of 19,414 background genes. GSEA was set to analyze biological processes related to AD&HD and FOXO1. The overlapping DEGs between the AD&HD/control and FOXO1-high/low groups were further analyzed by WGCNA. Coexpression gene modules were predicted for further functional enrichment analysis. A global regulation network was constructed to identify cross-talking pathways of FOXO1, thus exploring the potential mechanism of FOXO1 in these two diseases. Thenceforth, we applied the same method to screen out the cross-talking pathways of FOXO1 in AD and HD, respectively. AUC analysis was carried out to assess the diagnostic performance of FOXO1 in differentiating AD and HD from nondementia controls.

## 3. Results

### 3.1. Identification of DEGs

**Figure 2:**
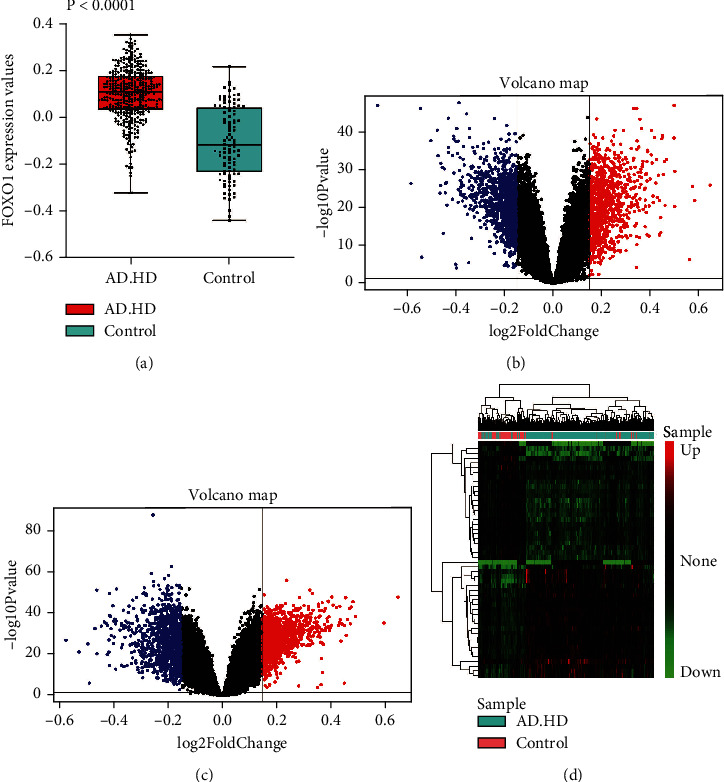
Differential expression gene analysis. FOXO1 expression between AD&HD and nondementia controls (a). Volcano plot of the AD&HD/control (b) and FOXO1-high/low group (c): blue, black, and red, respectively, indicate downregulated, nonsignificant, and upregulated DEGs. The heatmap of the top 25 down- and upregulated DEGs (d). AD: Alzheimer's disease; HD: Huntington's disease; DEGs: differential expression genes.

In the present study, 367 patients and 98 nondementia controls over the age of 60 were included for this computational analysis (Supplementary Table 1).  Figure 2(a) shows the comparison of mean expressions of FOXO1 between patients and controls. The expression of FOXO1 in the AD&HD group (0.10 ± 0.11) was significantly higher than those in controls (−0.11 ± 0.15) (*P* < 0.0001). After removal of unannotated or duplicated genes, 19,414 background genes were summarized for further differential expression analysis. A total of 2,103 genes were differentially expressed in AD&HD compared with nondementia controls. Among them, 1,001 DEGs were found to be significantly upregulated, while 1,102 were downregulated (Figure 2(b)). In subjects with high versus low expression of FOXO1, in total, 2,124 DEGs consisted of 1,089 up- and 1,035 downregulated genes were identified (Figure 2(c)). Of these, 1,657 DEGs were overlapped between the AD&HD/control and FOXO1-low/high groups. The cluster heatmap of the top 25 up- and downregulated overlapping DEGs is shown in Figure 2(d).

### 3.2. Coexpression Network Construction by WGCNA

**Figure 3:**
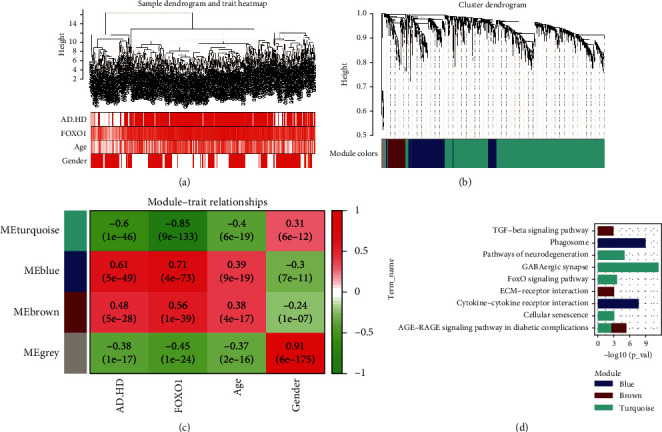
Weighted correlation network analysis. Sample dendrogram and trait heatmap (a). Four different coloured modules are used to form clustering dendrogram (b): grey stands for nonclustering genes. Heatmap of module-trait relationships (c): red indicates a positive correlation, green a negative correlation. Enrichment analysis of KEGG pathways for genes in coexpression modules (d). AD: Alzheimer's disease; HD: Huntington's disease; KEGG: Kyoto Encyclopedia of Genes and Genomes.

The set of DEGs was used for hierarchical clustering analysis and module-trait heatmap plotting. All samples passed the predefined cut-off line (height = 16) for the next step of bioinformatic analysis (Figure 3(a)). Using WGCNA, we predicted four coexpression modules with different colours based on the overlapping DEGs between the AD&HD/control and FOXO1-high/low groups (Figure 3(b)). As shown in the module-trait relationships (Figure 3(c)), the blue module of 344 DEGs had the strongest positive correlation with AD&HD (correlation coefficient = 0.61, *P* = 5*e* − 49) and FOXO1 (correlation coefficient = 0.71, *P* = 4*e* − 73); the turquoise module of 1,151 DEGs had the strongest negative correlation with AD&HD (correlation coefficient = −0.6, *P* = 1*e* − 46) and FOXO1 (correlation coefficient = −0.85, *P* = 9*e* − 133), while the brown module of 127 DEGs was positively correlated with AD&HD (correlation coefficient = 0.48, *P* = 5*e* − 28) and FOXO1 (correlation coefficient = 0.56, *P* = 1*e* − 39); and for the grey module, 35 noncoexpressed DEGs were clustered. These data suggest that DEGs in blue and turquoise modules had the strongest correlation with AD and HD.

### 3.3. Functional Enrichment Analysis of Coexpressed Modules

The mainly enriched KEGG pathways in the blue module were pathways of phagosome and cytokine-cytokine receptor interaction; brown module was enriched in transforming growth factor- (TGF-) *β* signaling pathway, extracellular matrix (ECM)-receptor interaction, and advanced glycation end product- (AGE-) receptors for AGEs (RAGE) signaling pathway in diabetic complications; for turquoise module, DEGs were involved in pathway of neurodegeneration, GABAergic synapse, FOXO signaling pathway, cellular senescence, and AGE -RAGE signaling pathway in diabetic complications (Figure 3(d)).

### 3.4. Construction of Module-Pathway Network of FOXO1

**Figure 4:**
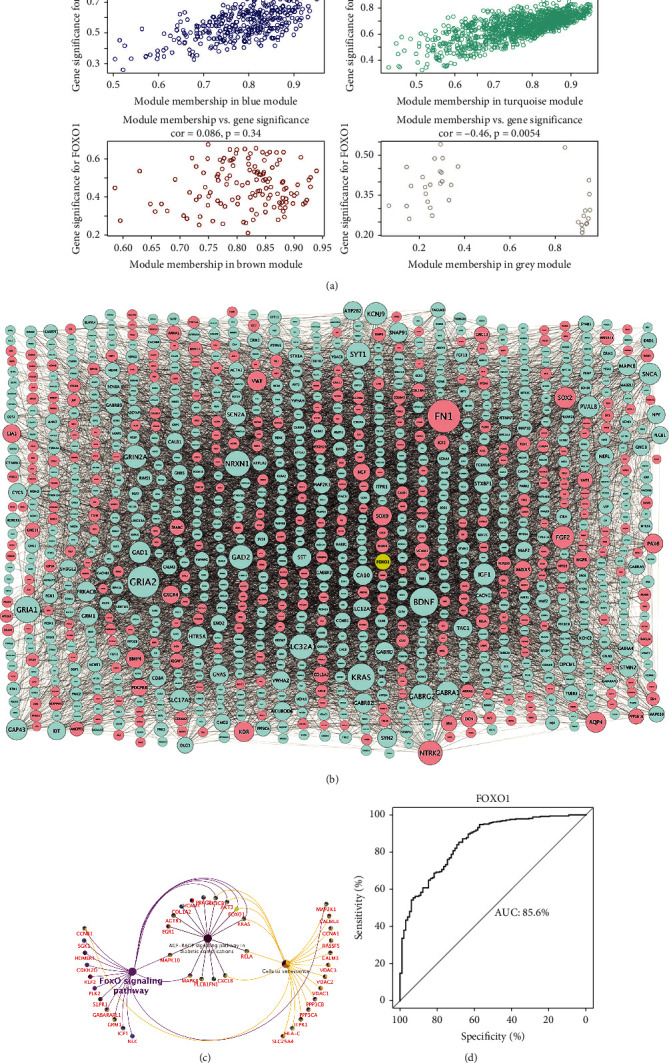
Module-pathway regulatory network of FOXO1 and AUC analysis. Scatterplot of module membership versus gene significance (a). Global regulatory network of turquoise and blue module (b): node size represents the degree of gene connectivity; blue represents low expression of the gene; yellow and red represent high expression. The cross-talking pathways of FOXO1 (c). Performance evaluation of AUC analysis (d). AUC: area under the curve.

**Figure 5:**
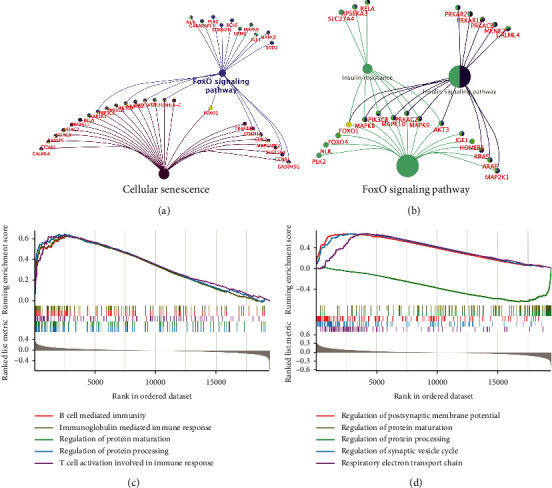
Module-pathway regulatory network of FOXO1 in single disease and gene set enrichment analysis. The pathways of FOXO1 enriched in AD (a) and HD (b). Biological processes enriched in AD&HD (c) and high expression of FOXO1 (d). AD: Alzheimer's disease; HD: Huntington's disease.

According to the scatter plot of relationship between GS and MM, DEGs in blue and turquoise modules showed the strongest correlation of intramodular connectivity with genetic phenotype (blue: correlation coefficient = 0.59, *P* = 1.2*e* − 33; turquoise: correlation coefficient = 0.73, *P* = 3.8*e* − 192) (Figure 4(a)). We extracted DEGs from the blue and turquoise modules and displayed them in the global regulatory network (Figure 4(b)). As shown in Figure 4(c), the cross-talking pathways of FOXO1 were identified, including cellular senescence, FOXO signaling pathway, and AGE-RAGE signaling pathway in diabetic complications. According to the AUC value of 85.6%, FOXO1 has potential predict value and may be a biomarker for AD and HD (Figure 4(d)). Separately, we found that the cross-pathways of FOXO1 in AD were related to FOXO signaling pathway and cellular senescence (Figure 5(a)); and the cross-pathways of FOXO1 in HD were linked to insulin resistance, insulin signaling pathway, and FOXO signaling pathway (Figure 5(b)).

### 3.5. GSEA Validation in Biological Processes

GSEA was adopted to validate the biological processes in the AD&HD and FOXO1-high groups. There were five biological processes significantly enriched in AD&HD, including B cell-mediated immunity, regulation of protein maturation, regulation of protein processing, immunoglobulin-mediated immune response, and T cell activation involved in immune response (Figure 5(c)). Five biological processes including regulation of postsynaptic membrane potential, regulation of protein maturation, regulation of protein processing, regulation of synaptic vesicle cycle, and respiratory electron transport chain were significantly enriched in the FOXO1-high group (Figure 5(d)). Of these, regulation of protein maturation and processing were both enriched in the AD&HD and FOXO1-high groups, suggesting that FOXO1 have implications in the onset and progression of these two diseases through protein synthesis.

## 4. Discussion

The GSEA results showed that the background genes in both AD&HD/control and FOXO1-high/low expression cohorts were enriched in biological processes of protein maturation and processing regulation. The newly synthesized proteins are processed in the endoplasmic reticulum (ER) to form mature proteins with physiological functions. In the presence of cellular crowding, gene mutations, oxidative stress, etc., protein processing in the ER is compromised, resulting in the formation of misfolded proteins. Subsequently, the accumulation of misfolded proteins leads to a state of ER stress to degrade these misfolded proteins. FOXO1 protein participates in such processes by modulating autophagy of misfolded proteins or apoptosis of impaired cells [31–33], consistent with our results of functional enrichment analysis. However, prolonged ER stress can also cause cell damage. It has been reported that ER stress in glial cells elicits the secretion of TNF-*α*, IL-1*β*, IL-6, and IL-8 [34]. These proinflammatory factors, in turn, facilitate the production of nitric oxide (NO) involving oxidative damage in glial cells [35]. Indeed, increased NO synthetase has been found in glia cells surrounding NFTs and amyloid deposition in AD brains [36], along with upregulated immunoreactivity in neurons adjacent to NO in these regions [37]. The mutant Huntingtin in cultured cells and neurons of postmortem HD brains exacerbates ER stress to impair ER-associated protein degradation (ERAD) early in the onset of HD [38–40]. Moreover, translocation of mutant Huntingtin into the nucleus is also inhibited, leading to persistent ER stress and long-term autophagic damage [41]. In addition, reduced organelle synthesis in ER that degrades misfolded proteins is also closely associated with the development of neurodegenerative diseases [42, 43]. It is reported that ER stress can be induced by mutations in the PSEN1 gene contributing consequently to dysfunctional lysosome synthesis, a cause potentially responsible for familial AD [44]. Therefore, these findings lend strong support to our notion that FOXO1 plays an essential role in protein processing and maturation and is closely associated with the pathology of proteotoxicity-related diseases such as AD and HD.

FOXO factors lie in the center of a complex regulatory network of multiple upstream pathways and downstream target genes, receiving upstream signals simultaneously or sequentially to regulate transcriptional activity of downstream target genes in normal or pathological cells. The PI3K/Akt/FOXO pathway is one of the major FOXO pathways that regulates the activation and localization of FOXO1. Knockdown of the upstream insulin receptor substrate (IRS) of this pathway leads to hyperactivation of FOXO1 [45]. Subsequently, activated FOXO1 disrupts mitochondrial oxidative and phosphorylation activities (OXPHOS), resulting in deficient ATP synthesis and metabolic disorders [46]. Notably, the resultant bioenergetic deficits in astrocytes and neurons are one of the most prevalent early features of AD [47]. This is in line with evidence in HD mouse models that mutant HTT (mHTT) aggregation recruits IRS-2 to activate FOXO1 via the PI3K/Akt/FOXO1 pathway, which contributes to mitochondrial dysfunction [48]. Furthermore, mHTT affects mitochondrial oxygenation to enhance anaerobic metabolism in the basal ganglia and hippocampus of HD patients, leading to increased levels of ROS; in turn, this process accelerates mitochondrial dysfunction and thus to form a vicious circle [49]. Several experiments have found a strong link of impaired insulin secretion and insulin resistance to HD. The incidence of diabetes in HD patients is seven times higher than that of normal diabetes, whose pathological features are reduced insulin secretion and increased insulin resistance. Moreover, even in HD patients with normal glycaemia, there is substantial insulin resistance [50]. Yamamoto et al. have demonstrated that activation of IRS-2 not only affects mitochondrial function but also leads to autophagy of accumulated mHTT proteins via the PI3K/Akt pathway, a branch of insulin signaling pathway highlighting the importance of insulin regulatory mechanisms for HD pathogenesis [47]. This is consistent with our results on the cross-talking pathways of FOXO1 in HD patients.

ROS are mainly produced by and act on mitochondria to regulate cell growth and differentiation at low concentrations [51]. JNKs belong to the mitogen-activated protein kinase (MAPK) family. The JNK pathway is predominantly activated by oxidative stress, and activation of phosphorylated substrates by JNK extensively induces apoptosis [52]. It is well known that aberrant accumulation of ROS leading to neuronal exposure to oxidative stress is a common feature of both AD and HD [53]. And a major consequence of this feature is an increase of cellular apoptosis. Specifically, MAPK kinase (MAP3K) is activated to form the JNK/FOXO1 signaling pathway, which induces neuronal apoptosis by nuclear translocation and phosphorylation of FOXO1 [54, 55]. In experiments of APP transgenic mouse brains, it has been shown that ROS-induced oxidative stress enhanced neuronal apoptosis [56]. Aggregation of A*β* induces excessive mitochondrial production of ROS; conversely, sustained activation of the JNK pathway induces increased expression of *β*-secretase and *γ*-secretase under conditions of oxidative stress, which in turn promotes A*β* production [57]. In HD pathology, mutant HTT increases the length of CAG repeats in neurons to overproduce mitochondrial ROS, which then activates the JNK pathway [58, 59], upregulating the expression of proapoptotic genes and thus to apoptosis [60, 61].

Following the process of apoptosis, the removal of apoptotic and senescent cells by phagocytosis contributes to the emergence of phagosomes, which are vesicles formed by the fusion of the cell membrane of the phagocyte around the granule. Fusion with lysosomes results in maturation of phagosome, which not only contains hydrolases and ROS to digest debris but also forms proinflammatory factors via activation of MAPK signaling and PI3K/Akt pathways [62]. Microglia, as special phagocytes of the central nervous system (CNS), recognize aggregated A*β* and mHTT, leading to a sustained release of neuroinflammatory factors for inflammatory damage and cell death [63]. Neuroinflammatory cell infiltration and microglia activation are inhibited through the PI3K/Akt/FOXO1 pathway, thus alleviating apoptosis and neurologic impairment after intracerebral hemorrhage [64]. Similarly, Chen et al. found that neurocognitive disorders could be ameliorated through the PI3K/Akt/FOXO1 pathway [65], consistent with our enrichment analysis of the blue module.

Autophagy is another essential mechanism for eliminating organelles in response to stress or starvation. It is involved in the phosphorylation of the downstream target FOXO1 in dynamic equilibrium through synergistic, inhibitory, or cross-talking action of the AMPK, PI3K/Akt, and JNK pathways [66]. Recent experiments have confirmed that JNK activation responds to oxidative stress by inhibiting FOXO-induced autophagy related gene expression, leading to reduced clearance of cell debris and aggregative proteins [67]. Thus, the JNK pathway plays a deleterious role in neurological impairment in neurodegenerative models by promoting apoptosis and inhibiting autophagy [68]. Under oxidative stress caused by aberrant protein synthesis, aggregation, or mitochondrial dysfunction, neurons or glial cells regulate nuclear translocation and activity of FOXO1 through PI3K/Akt and JNK pathways, hence mediating the pathological process of neurodegeneration. Likewise, these findings are in line with functional enrichment analyses of AD and HD obtained in combination or separately.

Oxidative stimulation acts on cellular components through different signaling pathways or exerts varying effects via the same pathways, determining diverse or even completely opposite cell fates. The precise mechanism is not fully understood, and the results of related experiments are variable and controversial. Salih and Brunet reported that apoptosis was the preferred cellular mechanism of FOXO1-activated neurons exposed to oxidative stress [69]. Nevertheless, Li et al. presented evidence that autophagy was initially triggered and only after prolonged stress could cells undergo apoptosis, which degraded aggregated proteins and damaged cells [70]. Hence, additional experiments are needed to prove the most realistic conclusion.

Cellular senescence participates in physiological processes including wound healing, tissue repair, and embryonic development, which is also a protective mechanism against tumor propagation triggered by oxidative stress-induced DNA damage or oncogenic signals. Moreover, it has also been observed that increased cellular senescence may contribute to senescence-associated diseases including ND [71]. Senescent cells alter proteostasis, promote the secretion of inflammatory cytokines, or allow the synthesis and aggregation of misfolded proteins, either of which ultimately leads to AD [72]. Cell senescence can also trigger iron accumulation, giving rise to oxidative death of neurons and glia cells associated with AD pathology [73]. Sirtuin 1 (SIRT1), a cellular senescence regulatory gene, has been shown to suppress FOXO1 expression in animal models of AD and HD, thereby attenuating neuronal degeneration and death [74, 75]. Additional evidence of *in vivo* HD model has also confirmed that SIRT1 activation provides a positive complement to mitochondrial failure, improving motor coordination and learning in HD [76].

The glycosylate modification of proteins by sugars and aldehydes in an oxygenated environment can produce AGEs [77]. AGEs binding to their receptors namely, RAGEs, promote the synthesis of inflammatory factors and the production of ROS, [78], leading to the accumulation of glycosylated proteins in different organs and tissues [79–81]. The coupling of AGE with RAGE and their interaction in the development of diabetes and its complications have been well established [82]. In the CNS, neurons exposed to oxidative stress also tend to form glycosylated proteins. Glycosylation of mitochondrial enzymes leads to disruption of energy transduction, affecting adenosine triphosphate (ATP) synthesis and subsequent biological processes [83]. The resultant deposition of glycosylated proteins utilizes ROS as second messengers to synthesize and bind inflammatory factors involved in HD and AD through the AGE-RAGE signaling pathway [84–86]. In fibroblasts, ROS generated via the AGE-RAGE signaling pathway acts on JNK to activate FOXO1, thus inducing apoptosis and preventing diabetic wound healing [87]. The vast majority of experiments on the role of FOXO1 in the AGE-RAGE signaling pathway have focused on diabetes and its complications. However, our findings suggest that this pathway may also be involved in AD and HD through the action of ROS and inflammatory factors. And the findings on endothelial cell integrity [87] may provide novel insights into investigating the integrity of cerebrovascular endothelium in relation to FOXO1-mediated AGE-RAGE signaling pathway in AD or HD.

Cumulating evidence suggested that GABAergic synaptic dysfunction aggravated cognitive impairment in patients with dementia [88–90]. Although few studies have linked GABAergic synapses to FOXO1, Zullo et al. found that GABAergic neurotransmitters and FOXO1 are regulated by the same transcription factors involved in life longevity [91]. Recently, GABA has been reported to be essential for the localization of DAF-16, a homologous transcription factor of the FOXO family in C. elegans, which triggers nuclear translocation by inhibiting insulin phosphorylation of FOXO via the DAF-2/IGF1R pathway [92].

The multifunctional TGF-*β* signaling pathway plays an important role in maintaining cellular homeostasis through apoptosis, autophagy, and cellular senescence in a variety of cells [93–95]. As a signal transducer, FOXO1 binds to Smad complex to activate the TGF-*β*/Smad signaling pathway, thus hampering neuronal growth and participating in the pathogenesis of AD [96, 97]. Alternatively, cellular senescence is regulated by the interaction of the TGF-*β*/Smad pathway with the PI3K/Akt/FOXO1 pathway [98]. Likewise, the TGF-*β* signaling pathway has implications in the pathology of neurodegeneration by interacting with the FOXO signaling pathway, which is consistent with the results of our functional enrichment analysis in the brown module.

ECM is a highly dynamic, continuously remodeling tissue with a specific structure [99]. Expression of collagen, a major component of ECM, is substantially upregulated in the subclinical and clinical phases of AD, which is associated with increased A*β* deposition [100]. In addition, our enrichment analysis has clarified that FOXO1 is associated with the regulation of ECM-receptor interaction pathway, in line with evidence of osteoarthritis for the involvement of aberrant FOXO1 expression in ECM-receptor interaction pathway [101].

**Figure 6:**
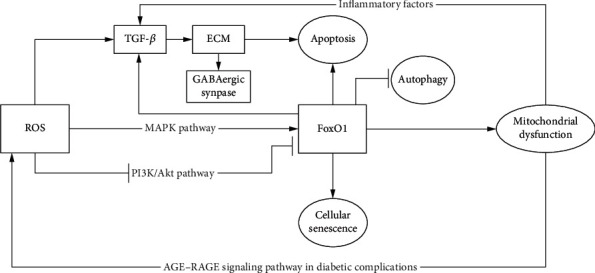
Cross-talking pathways of FOXO1 in AD and HD. AD: Alzheimer's disease; HD: Huntington's disease.

The onset and progression of AD or HD are the outcomes of the interaction of multiple mechanistic pathways. The cells are always in homeostasis by means of coordination, cross-regulation, and even mutual restraint among various pathways (Figure 6). For instance, AGE-RAGE interaction induces the release of TGF-*β* involving the synthesis of ECM proteins [102]. Furthermore, deposited glycosylated proteins modify the composition of ECM under oxidative stress, which triggers apoptosis of endothelial progenitor cells [103]. Additionally, previous studies in schizophrenia have demonstrated that abnormal EMC disturbs the connectivity of GABAergic synapses due to low expression of TGF-*β*1 [104, 105].

In the scatter plot between MM and GS, the strongest correlation between DEGs and FOXO1 expression was found in blue and turquoise modules. According to the cross-talking pathways of FOXO1 identified in the global regulatory network of DEGs, FOXO1 plays pleiotropic roles in the physiopathology of AD and HD via cellular senescence, FOXO signaling pathway, and AGE-RAGE signaling pathway in diabetic complications. The result of AUC analysis showed a good diagnostic performance in differentiating AD and HD patients from nondementia controls, which indicated that FOXO1 was possibly a predictive factor for the incidence of AD and HD. Collectively, the findings emerging from this study provide novel possible directions for experiments focusing on the timing and conditions whereby cells enter the senescence or apoptosis program and whether cerebrovascular endothelial integrity influences the progression of AD and HD through the interaction of FOXO1 and AGE-RAGE signaling pathways.

## 5. Conclusions

In summary, our findings support that the high expression of FOXO1 is responsible for the pathogenesis of AD and HD, possibly mediated by FOXO signaling pathway, cellular senescence, and the AGE-RAGE signaling pathway in diabetic complications.

## Data Availability

The data supporting this study is from previously reported studies and datasets, which are available in the GSE33000 repository of the GEO database.

## Supplementary Materials

Supplementary Materials Supplementary Table 1: clinical phenotypic data of enrolled samples.
