# Supplementary material for: Cross-Talking Pathways of Forkhead Box O1 (FOXO1) Are Involved in the Pathogenesis of Alzheimer's Disease and Huntington's Disease
Source: Oxid Med Cell Longev. 2022 Feb 3;2022:7619255. doi: 10.1155/2022/7619255 (PMC8831070; doi:10.1155/2022/7619255)
Supplement: Supplementary Materials — Supplementary Table 1: clinical phenotypic data of enrolled samples. [file 7619255.f1.pdf]

Sample	Group	Age	Gender	FOXO1
GSM1423780	AD	67	Female	-0.127339859
GSM1423781	AD	88	Male	0.008578354
GSM1423782	AD	62	Male	-0.213740803
GSM1423783	AD	90	Female	0.02778929
GSM1423784	AD	90	Female	-0.148348412
GSM1423785	AD	95	Female	0.121553438
GSM1423786	AD	77	Female	0.135600124
GSM1423787	AD	100	Female	0.169075128
GSM1423788	AD	72	Male	0.056688476
GSM1423789	AD	64	Female	0.233437375
GSM1423790	AD	89	Female	0.075266074
GSM1423791	AD	80	Female	0.244011632
GSM1423792	AD	61	Female	0.188573266
GSM1423793	AD	93	Male	0.117933579
GSM1423794	AD	81	Female	0.177987175
GSM1423795	AD	89	Male	0.239653338
GSM1423796	AD	81	Male	0.09964405
GSM1423797	AD	85	Female	0.023769824
GSM1423798	AD	79	Male	0.219616217
GSM1423799	AD	77	Female	0.223774587
GSM1423800	AD	91	Female	0.219672672
GSM1423801	AD	87	Male	-0.009824949
GSM1423802	AD	77	Female	0.171551504
GSM1423803	AD	77	Male	0.075812902
GSM1423804	AD	75	Female	0.102924898
GSM1423805	AD	76	Female	-0.056117952
GSM1423806	AD	84	Female	0.254961493
GSM1423807	AD	65	Male	0.26581474
GSM1423808	AD	66	Female	0.201747758
GSM1423810	AD	77	Male	0.260863437
GSM1423811	AD	61	Male	-0.030850342
GSM1423812	AD	79	Male	0.159469368
GSM1423813	AD	85	Male	0.02055705
GSM1423814	AD	79	Female	-0.037647505
GSM1423815	AD	89	Female	0.04348391
GSM1423816	AD	74	Male	0.019230994
GSM1423817	AD	89	Male	0.043696449
GSM1423818	AD	85	Female	0.097162171
GSM1423819	AD	67	Female	0.156966886

GSM1423820	AD	90	Female	0.350433884
GSM1423821	AD	92	Female	0.237851488
GSM1423822	AD	76	Female	-0.141427159
GSM1423823	AD	84	Female	0.208835584
GSM1423824	AD	83	Female	0.223576216
GSM1423825	AD	75	Female	-0.023517942
GSM1423826	AD	82	Male	0.185761079
GSM1423827	AD	87	Female	0.145846411
GSM1423828	AD	92	Male	0.187246851
GSM1423829	AD	74	Female	0.084314037
GSM1423830	AD	83	Female	-0.249455532
GSM1423831	AD	77	Male	0.086590631
GSM1423832	AD	68	Male	0.211740839
GSM1423833	AD	87	Male	0.014694986
GSM1423834	AD	83	Female	0.200568365
GSM1423835	AD	77	Male	0.14029233
GSM1423836	AD	78	Male	-0.019956713
GSM1423837	AD	85	Male	0.189828196
GSM1423838	AD	67	Female	0.029065464
GSM1423839	AD	89	Male	0.169413995
GSM1423840	AD	87	Female	0.166690484
GSM1423841	AD	74	Male	0.018044303
GSM1423842	AD	69	Female	0.218341327
GSM1423843	AD	73	Male	0.041830718
GSM1423844	AD	83	Male	0.12163156
GSM1423845	AD	62	Female	0.107619731
GSM1423846	AD	83	Male	0.120020606
GSM1423847	AD	75	Male	0.04321115
GSM1423848	AD	82	Male	0.102852321
GSM1423849	AD	83	Female	0.039763498
GSM1423850	AD	80	Male	0.21324122
GSM1423851	AD	92	Female	0.281694619
GSM1423852	AD	81	Female	-0.028247677
GSM1423853	AD	85	Female	0.293908996
GSM1423854	AD	68	Female	0.155308442
GSM1423855	AD	81	Female	0.190467825
GSM1423856	AD	84	Female	0.237550256
GSM1423857	AD	73	Male	0.322866044
GSM1423858	AD	98	Female	0.303965937
GSM1423859	AD	82	Female	0.29007361

GSM1423860	AD	86	Male	0.290467973
GSM1423861	AD	71	Male	0.144564401
GSM1423862	AD	86	Female	-0.051368426
GSM1423863	AD	80	Female	0.142642207
GSM1423864	AD	77	Male	0.072870784
GSM1423865	AD	83	Female	0.256690501
GSM1423866	AD	88	Male	0.063122933
GSM1423867	AD	64	Male	-0.074329775
GSM1423868	AD	70	Male	0.244503178
GSM1423869	AD	82	Female	0.127803454
GSM1423870	AD	67	Female	0.198053173
GSM1423871	AD	80	Male	0.147103939
GSM1423872	AD	80	Female	0.314528155
GSM1423873	AD	85	Female	0.170197616
GSM1423874	AD	86	Female	0.190538912
GSM1423875	AD	85	Male	0.081713716
GSM1423876	AD	88	Male	-0.014082492
GSM1423877	AD	89	Male	0.19482432
GSM1423878	AD	81	Male	0.133675088
GSM1423879	AD	91	Female	0.243281068
GSM1423880	AD	89	Female	-0.018537024
GSM1423881	AD	76	Female	-0.042481098
GSM1423882	AD	90	Female	0.031733024
GSM1423883	AD	87	Female	0.245027471
GSM1423884	AD	73	Male	-0.169704931
GSM1423885	AD	77	Male	0.285082557
GSM1423886	AD	84	Female	0.122352072
GSM1423887	AD	87	Male	0.129111778
GSM1423888	AD	78	Male	0.243923154
GSM1423889	AD	73	Male	0.072315391
GSM1423890	AD	71	Male	0.314735561
GSM1423891	AD	93	Female	0.310496269
GSM1423892	AD	91	Female	0.13671242
GSM1423893	AD	87	Female	-0.069915513
GSM1423894	AD	95	Female	0.152960884
GSM1423895	AD	82	Male	0.24865508
GSM1423896	AD	72	Male	0.253804709
GSM1423897	AD	80	Male	0.332973326
GSM1423898	AD	88	Female	0.301390801
GSM1423899	AD	91	Female	0.09737448

GSM1423900	AD	94	Female	0.190770814
GSM1423901	AD	84	Female	0.071624608
GSM1423902	AD	80	Female	-0.111991673
GSM1423903	AD	80	Female	0.212130355
GSM1423904	AD	86	Male	0.180363701
GSM1423905	AD	67	Male	-0.320077098
GSM1423906	AD	73	Male	0.141471526
GSM1423907	AD	86	Female	0.162116341
GSM1423908	AD	94	Female	0.019338671
GSM1423909	AD	85	Male	0.067162981
GSM1423910	AD	86	Female	0.171236263
GSM1423911	AD	78	Female	0.319137294
GSM1423912	AD	61	Male	0.142647489
GSM1423913	AD	89	Female	0.102370121
GSM1423914	AD	74	Female	0.123701939
GSM1423915	AD	86	Female	0.111989343
GSM1423916	AD	73	Male	0.253625663
GSM1423918	AD	73	Female	0.119497948
GSM1423919	AD	79	Male	0.122006106
GSM1423920	AD	84	Female	-0.076250411
GSM1423921	AD	83	Female	0.238158771
GSM1423922	AD	75	Female	0.165420482
GSM1423923	AD	82	Male	0.136220481
GSM1423924	AD	86	Male	0.184810802
GSM1423925	AD	75	Male	0.012366856
GSM1423927	AD	87	Female	0.227183867
GSM1423928	AD	75	Female	0.18677715
GSM1423929	AD	75	Female	0.24336305
GSM1423930	AD	76	Male	0.280993023
GSM1423931	AD	62	Female	0.190614673
GSM1423932	AD	67	Male	0.100790911
GSM1423933	AD	82	Female	0.198253287
GSM1423934	AD	86	Male	0.231497456
GSM1423935	AD	73	Male	0.151271679
GSM1423936	AD	84	Female	0.142197714
GSM1423937	AD	65	Female	0.129995356
GSM1423938	AD	61	Female	0.091193437
GSM1423939	AD	76	Male	0.075225763
GSM1423940	AD	98	Male	0.107904774
GSM1423941	AD	72	Male	0.093736418

GSM1423942	AD	92	Male	-0.025363611
GSM1423943	AD	87	Male	0.055307769
GSM1423944	AD	93	Female	0.095841554
GSM1423945	AD	71	Male	0.217991986
GSM1423946	AD	87	Female	0.229458519
GSM1423947	AD	82	Male	0.043249022
GSM1423948	AD	63	Male	0.08022658
GSM1423949	AD	88	Female	0.272885947
GSM1423950	AD	77	Male	-0.321193
GSM1423951	AD	77	Male	0.167423907
GSM1423952	AD	92	Female	0.06882137
GSM1423953	AD	93	Female	0.181727258
GSM1423954	AD	69	Female	0.131513427
GSM1423955	AD	89	Male	0.053332159
GSM1423956	AD	91	Female	0.146915541
GSM1423957	AD	84	Female	0.1130117
GSM1423958	AD	86	Male	0.06968187
GSM1423959	AD	89	Female	0.152334108
GSM1423960	AD	76	Female	-0.00264839
GSM1423961	AD	78	Female	0.172705063
GSM1423962	AD	70	Female	0.073334032
GSM1423963	AD	90	Female	0.072811622
GSM1423964	AD	72	Female	0.197114877
GSM1423965	AD	63	Female	0.025537315
GSM1423966	AD	98	Male	0.070949258
GSM1423967	AD	93	Male	-0.048908009
GSM1423968	AD	69	Male	0.020740281
GSM1423969	AD	80	Female	0.13076501
GSM1423970	AD	79	Male	-0.016863875
GSM1423971	AD	91	Female	0.122739812
GSM1423972	AD	88	Female	0.022292303
GSM1423973	AD	95	Female	-0.017396135
GSM1423974	AD	66	Female	0.102661228
GSM1423975	AD	85	Male	0.120080464
GSM1423976	AD	84	Female	0.017461646
GSM1423977	AD	97	Female	0.167977784
GSM1423978	AD	84	Male	0.044045033
GSM1423979	AD	84	Female	0.251570955
GSM1423980	AD	78	Female	-0.064463965
GSM1423981	AD	88	Female	0.132849953

GSM1423982	AD	89	Female	0.112004601
GSM1423983	AD	84	Female	0.08775425
GSM1423984	AD	84	Female	0.235651094
GSM1423985	AD	80	Male	-0.08206429
GSM1423986	AD	77	Male	0.168859428
GSM1423987	AD	93	Female	-0.001155536
GSM1423988	AD	88	Male	0.134728369
GSM1423989	AD	78	Female	0.250238609
GSM1423990	AD	77	Female	0.096570494
GSM1423991	AD	87	Female	0.17569025
GSM1423992	AD	88	Female	0.238704097
GSM1423993	AD	84	Male	-0.030322048
GSM1423994	AD	80	Male	0.1289315
GSM1423995	AD	83	Female	0.177225713
GSM1423996	AD	81	Female	0.163909988
GSM1423997	AD	83	Female	0.097859999
GSM1423998	AD	77	Female	0.139736988
GSM1423999	AD	81	Female	0.116621497
GSM1424000	AD	87	Male	0.040746336
GSM1424001	AD	87	Female	0.178682319
GSM1424002	AD	85	Female	0.216068408
GSM1424003	AD	78	Male	0.138960932
GSM1424004	AD	83	Female	0.075283248
GSM1424005	AD	87	Female	0.096359994
GSM1424006	AD	80	Female	0.175856414
GSM1424007	AD	92	Female	-0.006014661
GSM1424008	AD	88	Female	0.095290826
GSM1424009	AD	73	Male	0.027553893
GSM1424010	AD	72	Male	-0.237171513
GSM1424011	AD	94	Female	0.112984986
GSM1424012	AD	72	Male	0.109840894
GSM1424013	AD	88	Female	0.144370937
GSM1424014	AD	87	Male	0.200984809
GSM1424015	AD	73	Female	-0.068446221
GSM1424016	AD	80	Female	0.027762342
GSM1424017	AD	92	Male	0.166594407
GSM1424018	AD	91	Female	-0.085803115
GSM1424019	AD	86	Female	0.12560805
GSM1424020	AD	87	Male	0.052618974
GSM1424021	AD	90	Male	0.197222136

GSM1424022	AD	75	Male	0.228046926
GSM1424023	AD	69	Female	0.051061618
GSM1424024	AD	85	Male	0.08455695
GSM1424025	AD	79	Female	0.116862037
GSM1424026	AD	91	Female	0.135281139
GSM1424027	AD	91	Female	0.093083172
GSM1424028	AD	90	Female	0.062241084
GSM1424029	AD	64	Male	-0.009499139
GSM1424030	AD	72	Male	0.12545065
GSM1424031	AD	84	Female	0.048118946
GSM1424032	AD	84	Female	0.187977703
GSM1424033	AD	86	Female	0.06045243
GSM1424034	AD	60	Female	0.112738603
GSM1424035	AD	79	Female	0.196542169
GSM1424036	AD	86	Female	0.205095471
GSM1424037	AD	80	Male	0.141389905
GSM1424038	AD	78	Male	0.075217024
GSM1424039	AD	82	Male	0.185306966
GSM1424040	AD	78	Male	0.275436309
GSM1424041	AD	87	Male	0.159702834
GSM1424042	AD	80	Male	0.072351792
GSM1424043	AD	90	Female	-0.034133691
GSM1424045	AD	82	Male	0.040666173
GSM1424046	AD	72	Male	-0.145722265
GSM1424047	AD	73	Female	0.156682689
GSM1424048	AD	80	Female	0.162819851
GSM1424049	AD	75	Male	0.212000911
GSM1424050	AD	79	Female	0.071988712
GSM1424051	AD	79	Female	0.085059383
GSM1424052	AD	73	Male	0.140193578
GSM1424053	AD	69	Male	0.23901719
GSM1424054	AD	88	Female	-0.013421075
GSM1424055	AD	72	Male	0.188821314
GSM1424056	AD	78	Male	0.073121203
GSM1424057	AD	72	Female	0.104204829
GSM1424058	AD	76	Female	0.15261641
GSM1424059	AD	87	Female	-0.045535061
GSM1424060	AD	92	Female	0.220052651
GSM1424061	AD	80	Female	-9.80E-05
GSM1424062	AD	86	Female	0.048171555

GSM1424063	AD	98 Male	-0.196729496
GSM1424064	AD	78 Male	-0.213391383
GSM1424065	AD	84 Male	-0.100494942
GSM1424066	AD	79 Male	0.057212673
GSM1424067	AD	83 Female	0.023120103
GSM1424068	AD	85 Female	0.004679372
GSM1424069	AD	89 Male	-0.005647553
GSM1424070	AD	66 Male	0.082880861
GSM1424071	AD	71 Male	0.069735772
GSM1424072	AD	71 Male	0.101607878
GSM1424073	AD	65 Male	0.073743796
GSM1424074	AD	70 Male	-0.011320364
GSM1424075	AD	78 Male	-0.066793539
GSM1424076	AD	94 Female	0.141249747
GSM1424077	AD	83 Female	0.148136461
GSM1424078	AD	79 Female	0.076920282
GSM1424079	AD	76 Male	-0.020471719
GSM1424081	AD	87 Male	0.16553365
GSM1424082	AD	72 Male	0.07241021
GSM1424083	AD	80 Female	0.040089405
GSM1424084	AD	89 Female	0.039523344
GSM1424085	AD	79 Male	0.017427936
GSM1424086	AD	93 Female	-0.114621657
GSM1424087	AD	90 Female	0.163530483
GSM1424088	AD	91 Male	0.131192434
GSM1424089	AD	72 Male	0.052681749
GSM1424091	Control	64 Male	-0.291859618
GSM1424092	Control	95 Female	0.061593475
GSM1424095	Control	62 Female	0.127110562
GSM1424098	Control	67 Male	-0.161681074
GSM1424099	Control	60 Female	0.077308611
GSM1424100	Control	82 Male	-0.014936192
GSM1424104	Control	67 Male	-0.136135089
GSM1424106	Control	66 Male	-0.194671337
GSM1424109	Control	75 Female	0.113483985
GSM1424110	Control	68 Male	-0.14117942
GSM1424111	Control	60 Male	-0.229667009
GSM1424112	Control	61 Male	-0.27003573
GSM1424113	Control	74 Female	0.089105366
GSM1424114	Control	72 Male	-0.020937186

GSM1424115	Control	73	Male	-0.096152301
GSM1424118	Control	68	Male	-0.274901076
GSM1424119	Control	74	Female	0.002633538
GSM1424121	Control	69	Female	-0.170490077
GSM1424122	Control	64	Male	-0.265844104
GSM1424125	Control	72	Male	-0.147346981
GSM1424127	Control	60	Female	-0.045862628
GSM1424129	Control	72	Male	0.020521829
GSM1424130	Control	74	Male	-0.249149481
GSM1424131	Control	66	Female	0.047874981
GSM1424132	Control	70	Male	-0.035330489
GSM1424135	Control	65	Male	-0.437600442
GSM1424136	Control	75	Female	-0.14914158
GSM1424139	Control	61	Male	-0.289631066
GSM1424141	Control	62	Male	-0.177480983
GSM1424142	Control	65	Female	-0.023124179
GSM1424144	Control	75	Male	-0.324521384
GSM1424145	Control	68	Male	0.094799658
GSM1424146	Control	75	Male	-0.098921721
GSM1424148	Control	61	Male	-0.293889446
GSM1424149	Control	71	Male	-0.009589788
GSM1424150	Control	77	Male	-0.18195658
GSM1424152	Control	65	Female	0.147971742
GSM1424154	Control	106	Male	0.057684549
GSM1424156	Control	60	Male	-0.194525595
GSM1424157	Control	60	Male	-0.162533885
GSM1424161	Control	72	Female	0.074864608
GSM1424163	Control	70	Male	0.013313631
GSM1424165	Control	66	Male	-0.132717449
GSM1424166	Control	63	Female	-0.239886126
GSM1424167	Control	73	Male	0.026881004
GSM1424169	Control	74	Male	0.066428384
GSM1424170	Control	71	Female	-0.116107422
GSM1424172	Control	69	Female	0.073893545
GSM1424173	Control	78	Female	0.039845633
GSM1424174	Control	71	Male	-0.190814234
GSM1424175	Control	69	Male	0.045707409
GSM1424176	Control	60	Male	-0.395081976
GSM1424177	Control	61	Male	-0.420179496
GSM1424178	Control	62	Male	-0.148220038

GSM1424184	Control	66 Male	-0.214404353
GSM1424185	Control	80 Male	-0.041739851
GSM1424187	Control	69 Male	-0.222907216
GSM1424189	Control	61 Male	-0.283271672
GSM1424190	Control	65 Female	-0.270978858
GSM1424191	Control	60 Male	-0.350075923
GSM1424192	Control	72 Male	-0.171303008
GSM1424193	Control	63 Male	-0.343416345
GSM1424194	Control	76 Male	0.047719756
GSM1424195	Control	68 Male	-0.086169713
GSM1424196	Control	69 Female	0.041745012
GSM1424197	Control	63 Male	-0.20557554
GSM1424198	Control	74 Male	0.043663614
GSM1424200	Control	65 Male	-0.086398823
GSM1424201	Control	78 Female	-0.340760499
GSM1424202	Control	74 Male	-0.124355366
GSM1424203	Control	61 Male	-0.072776155
GSM1424204	Control	62 Male	-0.325830302
GSM1424205	Control	63 Male	-0.143140218
GSM1424206	Control	77 Male	-0.228766938
GSM1424208	Control	65 Male	0.096581303
GSM1424209	Control	64 Male	0.122569718
GSM1424210	Control	66 Male	0.074365157
GSM1424211	Control	86 Male	0.087973972
GSM1424213	Control	82 Male	0.137866621
GSM1424214	Control	71 Female	-0.02490422
GSM1424215	Control	73 Male	-0.014533231
GSM1424216	Control	68 Female	-0.112768288
GSM1424217	Control	75 Male	0.214404463
GSM1424218	Control	73 Female	0.029026281
GSM1424219	Control	79 Male	-0.143970581
GSM1424220	Control	68 Male	0.086055209
GSM1424222	Control	76 Male	-0.118553575
GSM1424225	Control	66 Male	-0.259159212
GSM1424226	Control	60 Male	-0.32885048
GSM1424227	Control	60 Male	-0.099861835
GSM1424231	Control	80 Male	-0.240984584
GSM1424232	Control	66 Male	-0.055384946
GSM1424235	Control	64 Male	-0.057449043
GSM1424238	Control	72 Male	-0.24165137

GSM1424241	Control	65	Male	-0.20167461
GSM1424243	Control	61	Male	-0.24242857
GSM1424244	Control	60	Female	0.04839474
GSM1424246	Control	62	Male	-0.307839037
GSM1424247	HD	60	Female	0.103017926
GSM1424249	HD	63	Male	0.025700283
GSM1424250	HD	81	Female	0.002108223
GSM1424252	HD	78	Female	0.013298491
GSM1424254	HD	75	Male	-0.20818485
GSM1424257	HD	63	Male	0.200329423
GSM1424260	HD	73	Male	0.043870533
GSM1424264	HD	73	Male	0.131819307
GSM1424265	HD	60	Female	-0.020602841
GSM1424266	HD	70	Male	0.154014425
GSM1424267	HD	74	Male	0.196321131
GSM1424268	HD	68	Female	0.098310527
GSM1424269	HD	74	Female	0.044366245
GSM1424271	HD	68	Male	0.158205446
GSM1424278	HD	62	Male	0.036969277
GSM1424283	HD	74	Male	0.136894927
GSM1424285	HD	60	Male	-0.024013253
GSM1424287	HD	74	Male	-0.033693831
GSM1424294	HD	62	Male	-0.040808121
GSM1424295	HD	83	Female	0.134458117
GSM1424297	HD	64	Male	0.017398165
GSM1424305	HD	69	Female	0.219117423
GSM1424307	HD	67	Female	0.16724079
GSM1424310	HD	63	Male	-0.018123663
GSM1424311	HD	67	Male	0.075796722
GSM1424312	HD	72	Female	-0.012994593
GSM1424313	HD	65	Female	0.038212916
GSM1424314	HD	72	Female	0.050152063
GSM1424316	HD	64	Male	0.10893011
GSM1424317	HD	64	Male	-0.085254344
GSM1424318	HD	61	Male	0.037328992
GSM1424325	HD	77	Male	0.1054369
GSM1424326	HD	83	Female	0.129739279
GSM1424327	HD	82	Female	0.172250817
GSM1424329	HD	73	Female	0.009746321
GSM1424330	HD	75	Female	-0.057236033

GSM1424331	HD	82 Male	0.200187482
GSM1424332	HD	85 Female	0.076283794
GSM1424333	HD	60 Male	-0.116646812
GSM1424336	HD	68 Female	0.031670884
GSM1424338	HD	80 Male	0.042552077
GSM1424339	HD	84 Male	0.120992364
GSM1424341	HD	73 Male	0.076312452
GSM1424342	HD	81 Male	0.029863132
GSM1424348	HD	62 Male	0.128291103
GSM1424355	HD	66 Male	-0.085963285
GSM1424356	HD	64 Male	0.06119008
GSM1424357	HD	75 Male	-0.029811698
GSM1424358	HD	60 Female	0.267670464
GSM1424359	HD	77 Female	0.125739984
GSM1424366	HD	70 Male	0.042749192
GSM1424367	HD	67 Male	0.065218277
GSM1424369	HD	66 Male	-0.066481121
GSM1424378	HD	74 Male	0.143528669
GSM1424379	HD	65 Male	0.053823795
GSM1424380	HD	66 Female	-0.091166761
GSM1424384	HD	72 Male	0.041549321
GSM1424388	HD	72 Male	0.02927982
GSM1424392	HD	75 Male	0.135680994
GSM1424393	HD	80 Female	-0.136433022
GSM1424398	HD	69 Female	0.047664394
GSM1424399	HD	64 Male	0.124906614
